# Mental health prevalence and predictors among university students in nine countries during the COVID-19 pandemic: a cross-national study

**DOI:** 10.1038/s41598-021-97697-3

**Published:** 2021-09-20

**Authors:** Dominika Ochnik, Aleksandra M. Rogowska, Cezary Kuśnierz, Monika Jakubiak, Astrid Schütz, Marco J. Held, Ana Arzenšek, Joy Benatov, Rony Berger, Elena V. Korchagina, Iuliia Pavlova, Ivana Blažková, Imran Aslan, Orhan Çınar, Yonni Angel Cuero-Acosta

**Affiliations:** 1 Faculty of Medicine, University of Technology, 40-555 Katowice, Poland; 2 Institute of Psychology, University of Opole, 45-052 Opole, Poland; 3 Faculty of Physical Education and Physiotherapy, Opole University of Technology, 45-758 Opole, Poland; 4 Faculty of Economics, Maria Curie-Sklodowska University in Lublin, 20-031 Lublin, Poland; 5 Department of Psychology, University of Bamberg, 96047 Bamberg, Germany; 6 Faculty of Management, University of Primorska, 6101 Koper, Slovenia; 7 Department of Special Education, University of Haifa, 3498838 Haifa, Israel; 8 The Center for Compassionate Mindful Education, 69106 Tel Aviv, Israel; 9 Bob Shapell School of Social Work, Tel-Aviv University, 69978 Tel Aviv, Israel; 10 Institute of Industrial Management, Economics and Trade, Peter the Great St. Petersburg Polytechnic University, St. Petersburg, 195251 Russia; 11 Department of Theory and Methods of Physical Culture, Lviv State University of Physical Culture, Lviv, 79007 Ukraine; 12 Department of Regional and Business Economics, Mendel University in Brno, Brno, 613 00 Czech Republic; 13 Health Management Department, Bingöl University, Bingöl, 12000 Turkey; 14 Faculty of Economics and Administrative Sciences, Ataturk University, Erzurum, 25240 Turkey; 15 Faculty of Economics and Administrative Sciences, Ağrı İbrahim Çeçen University, Ağrı, 04000 Turkey; 16 School of Management, Universidad del Rosario, 111711 Bogotá, PC Colombia

**Keywords:** Quality of life, Population screening

## Abstract

The student population has been highly vulnerable to the risk of mental health deterioration during the coronavirus disease (COVID-19) pandemic. This study aimed to reveal the prevalence and predictors of mental health among students in Poland, Slovenia, Czechia, Ukraine, Russia, Germany, Turkey, Israel, and Colombia in a socioeconomic context during the COVID-19 pandemic. The study was conducted among 2349 students (69% women) from May–July 2020. Data were collected by means of the Generalized Anxiety Disorder (GAD-7), Patient Health Questionnaire (PHQ-8), Perceived Stress Scale (PSS-10), Gender Inequality Index (GII), Standard & Poor's Global Ratings, the Oxford COVID-19 Government Response Tracker (OxCGRT), and a sociodemographic survey. Descriptive statistics and Bayesian multilevel skew-normal regression analyses were conducted. The prevalence of high stress, depression, and generalized anxiety symptoms in the total sample was 61.30%, 40.3%, and 30%, respectively. The multilevel Bayesian model showed that female sex was a credible predictor of PSS-10, GAD-7, and PHQ-8 scores. In addition, place of residence (town) and educational level (first-cycle studies) were risk factors for the PHQ-8. This study showed that mental health issues are alarming in the student population. Regular psychological support should be provided to students by universities.

## Introduction

The coronavirus disease (COVID-19) pandemic is an unexpected, global phenomenon that has affected people worldwide in various aspects of life. Severe acute respiratory syndrome coronavirus-2 (SARS-CoV-2) is the virus responsible for the COVID-19 pandemic^1^ which affects the respiratory and central nervous systems system^2^. Apart from physical health, the ongoing pandemic has substantially affected mental health in a negative manner^3,4^. Due to preventive restrictions, the global population has led to social isolation on an unprecedented scale, which is strongly related to psychological distress, high anxiety, and acute stress^5–9^.

Even though young adults are the least susceptible to COVID-19 infection^10^, this group is the most vulnerable to mental health deterioration^11–13^. Young adults with a student status often deal with mental health issues. Even in the prepandemic period, more than one-third of students experienced mental health problems^14^.

Additionally, high stress and anxiety risk prevalence were observed in students prior to the pandemic^15^. Students are also at greater risk of depression than the general population^16^ or other types of occupational status, e.g., employed or retired^17^, including academic staff^18^. The coronavirus pandemic has affected students' lives in many aspects, i.e., distance learning, labor market, career opportunities or hygiene-related behavior, and daily routines^19^. Considering that mental health issues at a young age can lead to low employment rates, poor academic outcomes, and substantial loss of total earnings over the lifetime^20^, there should be a strong focus on research concerning students' mental health during the ongoing pandemic.

Even though the number of research papers referring to the COVID-19 pandemic has already exceeded the number of studies dedicated to Ebola and H1N1, few studies have emerged as the product of international collaboration^21^. Therefore, the pursuit of international studies is crucial to determine implications of the sociocultural context of mental health issues during the global pandemic.

Cross-national research on mental health during the COVID-19 pandemic more frequently pertains to the general population^22–26^ rather than the student population^27–29^. Evidence from 78 countries revealed that the prevalence of depressive symptoms was 25% in the general population and that of high stress was 11%^26^. A cross-national study showed that the prevalence of the depressive disorder was 30.7% for the general sample, with Czech students presenting lower levels of depression than Emirati students, but similar to the American and Taiwanese samples^28^. Israeli and Russian students presented a similar prevalence level concerning the feeling of being depressed: 45.3% and 46.4%, respectively^29^. The prevalence of moderate and severe anxiety symptoms among Polish students was 21% and 14%, respectively, and high perceived stress was manifested by 56% at the beginning of the pandemic^30^. In the student sample from Bavarian universities, nearly 40% reported an increased psychological burden, while 17.3% of the students claimed to experience less mental stress during the COVID-19 pandemic^31^.

Mental health prevalence varies in between-country comparisons as well as within a country. In China, the prevalence of anxiety symptoms in the adult population ranges from 6.33 to 35.1%, and depression symptoms range from 14.6 to 48.6%^32^. The differences might be due to the stage of the pandemic or measurements applied in the study. Another issue is the variety of sample sizes in cross-national research. In one study, it ranged from 33 (Chinese sample) to 869 respondents (USA sample)^22^ or from 1285 students in Latvia to 100 in the UK^26^. Additionally, it should be be noted that little attention has been devoted to understanding cross-national differences.

Several risk factors have contributed to declining mental health during the COVID-19 pandemic, including (female) gender, (younger) age, and (lower) income^24,33,34^. Even in research regarding the global population, student status appears to be a risk factor of mental health issues. This is especially valid for students of the first-cycle studies^19,23,26,34^. However, when analyzing the student population, the evidence behind risk factors found in the general population has been inconsistent. Age as a risk factor is noticeable among students. It was noted that being under 24 was linked to higher anxiety and depression^35^ and higher COVID-19-related psychological impact^36^. However, research among German students has shown that younger students (17–25) reported lower mental stress than older groups^31^. Female gender is considered a risk factor for students' mental health^30,31,35–37^. Nevertheless, male students reported a higher prevalence of depression and anxiety compared to female students in Bangladesh^38^. In Turkish students, gender was a weaker predictor of high perceived stress levels than the level of physical inactivity, and the role of gender was diminished by satisfaction with life and anxiety^39^. The role of the place of residence is unclear and depends on the country of origin. Living in an urban area was linked to lower anxiety in China^40^ but to higher anxiety and depression in Bangladesh^38^.

The nine countries in our study represent the cultural diversity portrayed by traditional vs. secular and survival vs. self-expression values. The Inglehart-Welzel World Cultural Map^41^ aggregates all countries into eight clusters based on those values. Six of the value clusters are exemplified in our study. Catholic Europe is represented by Poland, Slovenia, and Czechia; Orthodox Europe by Ukraine and Russia; Protestant Europe by Germany; African-Islamic region by Turkey; West and South Asia by Israel; and Latin America by Colombia. Therefore, these countries represent a great diversity of global cultural values.

In addition to cross-cultural differences, one possible explanation for the prevalence of mental health problemsin cross-national samples may be socioeconomic development indices, such as the Gender Inequality Index (GII)^42^, or the credit risk of the country as measured by Standard and Poor's Global Ratings (S&P)^43^. The COVID-19 pandemic has negatively affected the global economy on an unprecedented scale. It is perceived as the deepest global recession in eight decades^44^. Previous financial crises exposed increased levels of anxiety, depression^45^, and psychological stress^46^. Recession leads to higher mortality and suicide rates^47^. In addition, considering that lower social status is related to mental health deterioration^48,49^, it seems crucial to include the financial index in the mental health model. The S&P rating refers to the evaluation of credit risk at the national level. The index is more comprehensive compared to the gross domestic product (GDP) per capita, as it refers to the social situation^43^.

Additionally, during the pandemic, labor markets have fallen into a recession. Moreover, the existing gender gap has widened^50^. In light of the this, we propose applying the GII in this context. The GII is a comprehensive indicator referring to the dimensions of reproductive health (maternal mortality and adolescent birth ratio), empowerment (education, parliamentary seats), and labor market. The index relates to the Human Development Index (HDI). The greater the GII value, the more gaps between women and men exist, and the greater loss to human development. Both the GII and S&P credit ratings are well established and considered to be objective and reliable measurements.

**Figure 1:**
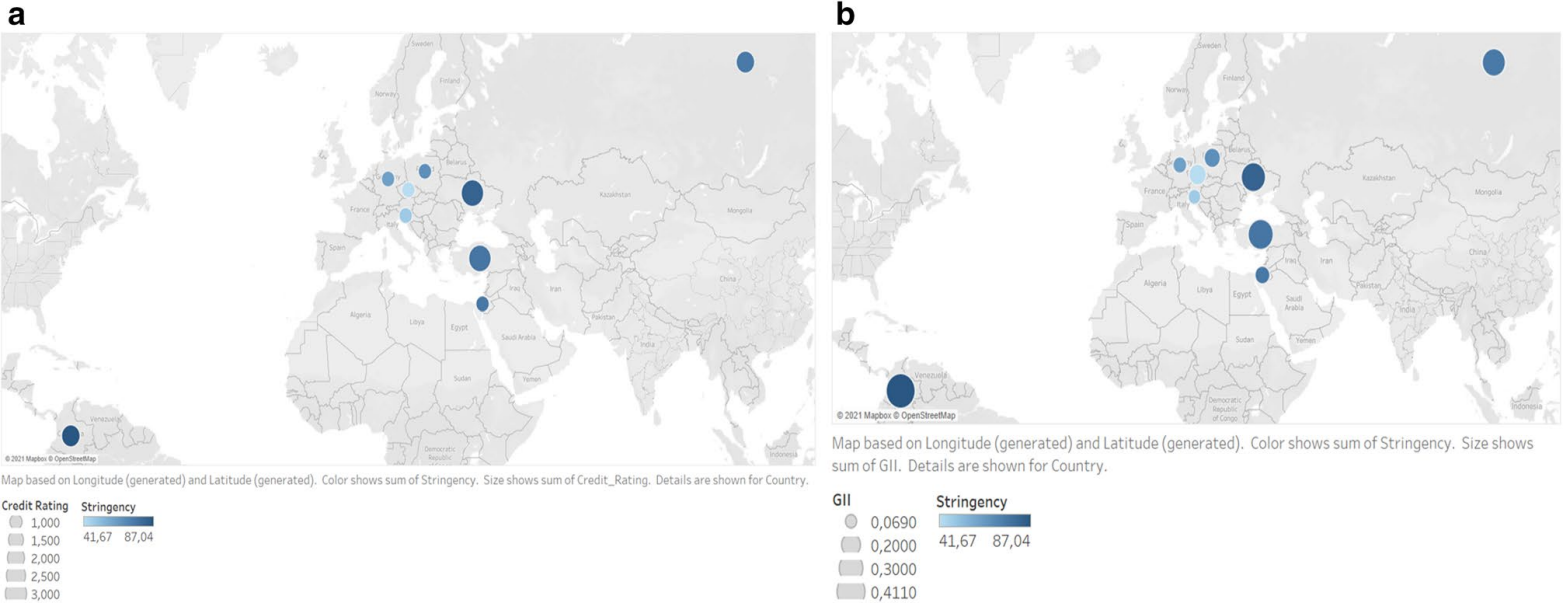
Maps present the following data in nine countries (Poland, Slovenia, Czechia, Ukraine, Russia, Germany, Turkey, Israel, and Colombia): (**a**) credit rating (indicated by the size of the dot) and the stringency level of restrictions (highlighted in color); (**b**) gender Inequality Index (GII) (size) and the stringency level (color). The map was developed in Tableau 2021.1.

To monitor the effect of national governmental restrictions on mental health, the Oxford COVID-19 Government Response Tracker (OxCGRT) was introduced. It enables the measurement of the stringency level of restrictions^51^, whichis based on three groups of indicators: community mobility, economic aspects and public health. This is rescaled to a value ranging from 0 to 100, where 100 denotes the strictest restrictions. During data collection, the stringency level varied across Poland, Slovenia, Czechia, Ukraine, Russia, Germany, Turkey, Israel, and Colombia, from the lowest in Czechia (41.67) to the highest in Colombia (87.04) (detailed analysis in Supplementary Table S1). The stringency of restrictions affects mental health as a study in six countries shows, although its role is rather limited^22^. The aforementioned macrolevel indices pertaining to the nine countries selected for the present study (Poland, Slovenia, Czechia, Ukraine, Russia, Germany, Turkey, Israel, and Colombia) are outlined in detail in Table S1. All countries differ regarding their socioeconomic situation as measured by the GII and S&P credit rating as well as the course of the pandemic indicated by means of the stringency level. The indexes are presented on geographical maps (Fig. 1) and reflect the situation at the moment of the study (May–July 2020). The aforementioned socioeconomic indices (GII and S&P Global Rating), together with the stringency level, have rarely been analyzed regarding mental health during the COVID-19 pandemic.

Our aim in this study was to reveal the prevalence and predictors of mental health among students in Poland, Slovenia, Czechia, Ukraine, Russia, Germany, Turkey, Israel, and Colombia in a socioeconomic context marked by the COVID-19 pandemic.

We will introduce a Bayesian multilevel prediction model combining macrolevel and individual-level variables. We will examine whether the individual-level (gender, place of residence, level of study) and macrolevel variables (stringency, GII, and S&P Rating) constitute predictors of anxiety, depression, and perceived stress at the subnational level among students in the nine countries during the COVID-19 pandemic.

We hypothesize that female gender, residence in a city, higher education level, higher stringency, and greater GII and S&P credit risk rating will constitute risk factors for all the measured mental health dimensions.

## Results

### Participants

The initial total sample was 2453 individuals. However, 104 persons (4.24% of the total sample) declined to participate (responded *No* to the informed consent). Therefore, the final total sample of university students participating in the study was 2349. All the respondents were eligible for the study and confirmed their student status. Additionally, respondents who did not want to reveal their gender were excluded from statistical analyses concerning gender (n = 6). A sample size was computed by G*Power software^52^ with regard to χ^2^ contingency tables, with *p* < 0.05, two-tailed, and 95% *CI*, and a sample size of 159 for each country was established.

### Descriptive statistics

The research sample consisted of 2349 students from Poland (n = 301), Slovenia (n = 209), Czechia (n = 310), Ukraine (n = 310), Russia (n = 285), Germany (n = 270), Turkey (n = 310), Israel (n = 199) and Colombia (n = 155). Women comprised 69.30% of the total sample, ranging from 55.80% in Turkey to 85.20% in Slovenia. Polish, Slovenian, and Czech students mostly lived in rural areas, Ukrainian and German in towns, Russian and Israeli in cities, whereas Turkish and Colombian students in large urban agglomerations. Most of the students attended first cycle studies (Bachelor, 78.50%) and studied full-time (85.40%). Detailed sociodemographic descriptive statistics are presented in Table 1. Comprehensive description of the recruitment (universities in each of nine countries) are presented in the Supplementary Note.

**Table 1:** Demographic characteristics of the study sample in the nine countries.

Demographic variables	Total N = 2349		Poland N = 301		Slovenia N = 209		Czechia N = 310		Ukraine N = 310		Russia N = 285		Germany N = 270		Turkey N = 310		Israel N = 199		Colombia N = 155	
	n	%	n	%	n	%	n	%	n	%	n	%	n	%	n	%	n	%	n	%
**Gender**																				
Women	1627	69.30	221	73.40	178	85.20	204	65.80	217	70.00	191	67.00	193	71.50	173	55.80	149	74.90	101	65.20
Men	710	30.20	80	26.60	31	14.80	104	33.50	93	30.00	92	32.20	77	27.80	133	42.90	50	25.10	52	33.50
Did not want to say	6	0.30	0	0.00	0	0.00	0	0.00	0	0.00	0	0.00	0	0.00	4	1.30	0	0.00	2	1.30
**Place of residence**																				
Village	614	26.10	141	46.80	85	40.70	127	41.00	112	36.10	7	2.50	53	19.60	52	16.80	37	18.60	0	0.00
Town	670	28.50	94	31.20	65	31.10	97	31.30	114	36.80	81	28.40	177	65.60	41	13.20	1	0.50	0	0.00
City	722	30.70	61	20.30	40	31.10	80	25.80	75	24.20	171	60.00	33	12.20	101	32.60	161	80.90	0	0.00
Agglomeration	343	14.60	5	1.70	19	9.10	6	1.90	9	2.90	26	9.10	7	2.60	116	37.40	0	0.00	155	100.00
**Level of study**																				
Bachelor	1843	78.50	171	56.80	143	68.40	226	72.90	291	93.90	245	86.10	137	50.70	283	91.30	196	98.50	151	97.40
Master	427	18.20	130	43.20	61	29.20	83	26.80	19	6.10	33	11.60	96	36.60	1	0.30	0	0.00	4	2.60
Postgraduate	67	2.90	0	0.00	0	0.00	0	0.00	0	0.00	7	2.50	35	13.00	25	8.10	0	0.00	0	0.00
Doctoral	12	0.50	0	0.00	5	2.40	1	0.30	0	0.00	0	0.00	2	0.70	1	0.30	3	1.50	0	0.00
**Type of study**																				
Full-time	2007	85.40	273	90.70	209	100.00	269	86.80	297	95.80	234	82.10	270	100.00	310	100.00	199	100.00	155	100.00
Part-time	342	14.60	28	9.30	0	0.00	41	13.20	13	4.20	51	17.90	0	0.00	0	0.00	0	0.00	0	0.00

**Figure 2:**
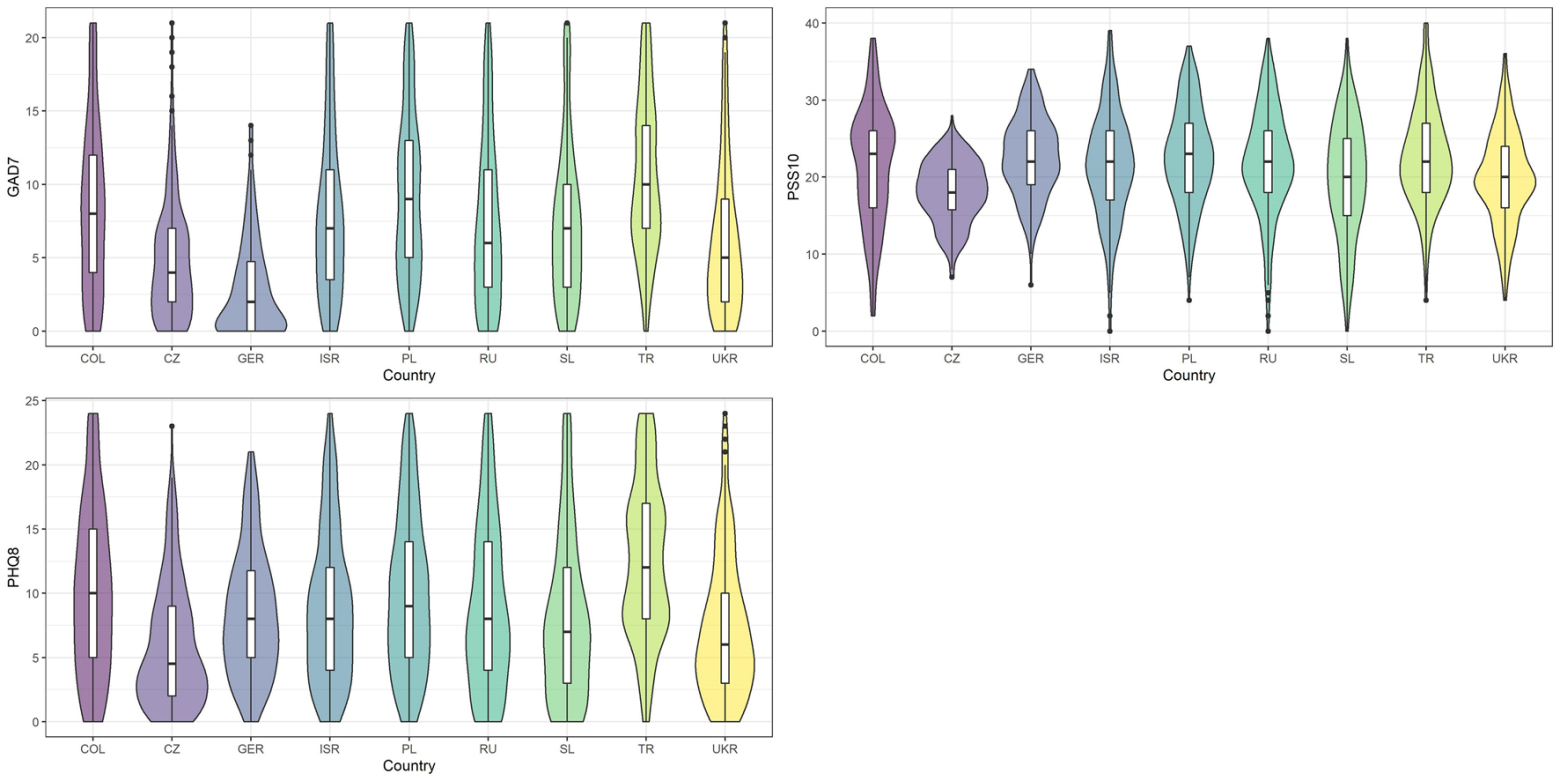
Distribution of anxiety (GAD-7), perceived stress (PSS-10), and depression (PHQ-8) parameters within each surveyed country presented by violin and box plots. COL = Colombia, CZ = Czechia, GER = Germany, ISR = Israel, PL = Poland, RU = Russia, SL = Slovenia, TR = Turkey, UKR = Ukraine.

The distribution of the generalized anxiety disorder (GAD-7), perceived stress (PSS-10), and depression (PHQ-8) scores in the nine countries is outlined in Fig. 2. There were no missing data in the statistical analyses. A comprehensive description is given in Table 2.

**Table 2:** Descriptive statistics of perceived stress (PSS-10), anxiety (GAD-7), and depression (PHQ-8) in nine countries (N = 2349).

Descriptive statistics		Perceived stress PSS-10	Anxiety GAD-7	Depression PHQ-8
Country	Range	0–40	0–21	0–24
Poland	M (SD)	22.69 (6.33)	9.20 (5.49)	9.99 (6.08)
	95% CI LL–95% CI UL	21.97–23.41	8.57–9.81	9.29–10.68
Slovenia	M (SD)	19.83 (7.56)	7.37 (5.33)	7.98 (6.27)
	95% CI LL–95% CI UL	18.80–20.86	6.65–8.10	7.13–8.84
Czechia	M (SD)	18.16 (3.99)	4.86 (3.98)	5.90 (4.70)
	95% CI LL–95% CI UL	17.71–18.60	4.42–5.31	5.38–6.43
Ukraine	M (SD)	19.93 (5.99)	6.15 (5.11)	7.32 (5.39)
	95% CI LL–95% CI UL	19.26–20.59	5.58–6.72	6.72–7.92
Russia	M (SD)	21.98 (6.95)	7.48 (5.70)	9.00 (6.41)
	95% CI LL–95% CI UL	21.17–22.79	6.82–8.14	8.25–9.75
Germany	M (SD)	22.54 (5.05)	2.92 (3.26)	8.42 (4.89)
	95% CI LL–95% CI UL	21.93–23.15	2.52–3.31	7.83–9.01
Turkey	M (SD)	22.71 (6.43)	10.41 (5.25)	12.42 (6.02)
	95% CI LL–95% CI UL	21.99–23.42	9.82–11.00	11.74–13.09
Israel	M (SD)	21.51 (6.88)	7.92 (5.56)	8.94 (5.81)
	95% CI LL–95% CI UL	20.54–22.47	7.14–8.70	8.13–9.75
Colombia	M (SD)	21.37 (7.55)	8.45 (5.79)	10.13 (6.41)
	95% CI LL–95% CI UL	20.17–22.57	7.53–9.37	9.11–11.15
Total	M (SD)	21.16 (6.44)	7.16 (5.52)	8.85 (6.05)
	95% CI LL–95% CI UL	20.90–21.42	6.49–7.39	8.60–9.10

M: mean; SD: standard deviation; CI: confidence interval, LL: lower limit of the confidence interval, UL: upper limit of the confidence interval.

### Prevalence of anxiety, depression, and perceived stress

The prevalence of mental health indicators in students from the nine countries is presented in Table 3. More than half of the respondents (61.30%) reported high PSS-10. Polish (71.10%) and Turkish (70.30%) students reported the greatest prevalence of high PSS-10, whereas Czech students (30%) reported the lowest prevalence.

**Table 3:** Prevalence of perceived stress (PSS-10), anxiety (GAD-7), and depression (PHQ-8) among students in nine countries.

Variable	Total *N* = 2349		Poland *N* = 301		Slovenia *N* = 209		Czechia *N* = 310		Ukraine *N* = 310		Russia *N* = 285		Germany *N* = 270		Turkey *N* = 310		Israel *N* = 199		Colombia *N* = 155	
	*n*	%	*n*	%	*n*	%	*n*	%	*n*	%	*n*	%	*n*	%	*n*	%	*n*	%	*n*	%
**Perceived stress (PSS-10)**																				
Low (0–13)	270	11.50	24	8.00	41	19.60	47	15.20	47	15.20	32	11.20	8	3.00	20	6.50	24	12.10	27	17.40
Medium (14–19)	639	27.20	63	20.90	54	25.80	141	45.50	101	32.60	62	21.80	65	24.10	72	23.20	46	23.10	35	22.60
High (20–40)	1440	61.30	214	71.10	114	54.50	122	39.40	162	52.30	191	67.00	197	73.00	218	70.30	129	64.80	93	60.00
**Anxiety (GAD-7)**																				
Normal (0–4)	905	38.50	71	23.60	71	34.00	164	52.90	142	45.80	103	36.10	202	74.80	40	12.90	64	32.20	48	31.00
Mild (5–9)	740	31.50	89	29.60	80	38.30	106	34.20	97	31.30	86	30.20	54	20.00	111	35.80	70	35.20	47	30.30
Moderate (10–14)	423	18.00	86	28.60	35	16.70	32	10.30	44	14.20	53	18.60	14	5.20	87	28.10	36	18.10	36	23.20
Severe (15–21)	281	12.00	55	18.30	23	11.00	8	2.60	27	8.70	43	15.10	0	0.00	72	23.20	29	14.60	24	15.50
**Depression (PHQ-8)**																				
Normal (0–4)	662	28.20	68	22.60	72	34.40	155	50.00	110	35.50	79	27.70	63	23.30	29	9.40	52	26.10	34	21.90
Mild (5–9)	738	31.40	85	28.20	67	32.10	90	29.00	110	35.50	89	31.20	102	37.80	88	28.40	63	31.70	44	28.40
Moderate (10–14)	483	20.60	76	25.20	34	16.30	44	14.20	52	16.80	58	20.40	68	25.20	70	22.60	43	21.60	38	24.50
Moderately severe (15–19)	305	13.00	46	15.30	21	10.00	20	6.50	25	8.10	36	12.60	29	10.70	77	24.80	26	13.10	25	16.10
Severe (20–24)	158	6.70	26	8.60	15	7.20	1	0.30	13	4.20	23	8.10	5	1.90	46	14.80	15	7.50	14	9.00
Anxiety symptoms (GAD-7 ≥ 10)	704	30.00	141	46.80	58	27.80	40	12.90	71	22.90	96	33.70	14	5.20	159	51.30	65	32.70	60	38.70
Depression symptoms (PHQ-8 ≥ 10)	946	40.30	148	49.20	70	33.50	65	21.00	90	29.00	117	41.10	102	37.80	193	62.30	84	42.20	77	49.70
Anxiety × depression symptoms	575	24.50	115	38.20	42	21.10	26	8.40	59	19.00	76	26.70	13	4.80	139	44.80	53	26.60	52	33.50
No symptoms	1271	54.10	127	42.20	123	58.90	231	74.50	208	67.10	148	51.90	164	60.70	97	31.30	103	51.80	70	45.20

The GAD-7 and PHQ-8 risks were reported in 30% and 40.30% of the total student sample, respectively. The highest prevalence of the GAD-7 and PHQ-8 risks occurred in Turkey, at 51.30% and 62.30%, respectively. However, the GAD-7 risk was also high in Poland (46.80%). The lowest occurrence of the GAD-7 risk emerged in Germany (5.20%) and the PHQ-8 emerged in Czechia (21%). Almost every fourth student (24.50%) in the nine countries experienced the GAD-7 and PHQ-8 risks, with the highest prevalence in Turkey (44.80%) and the lowest in Germany (4.80%).

### Bayesian multilevel regression analyses

Bayesian multilevel skew-normal regression analyses with a country as a grouping variable (i.e., 'random effect') were employed to reveal predictors of dependent variables: generalized anxiety disorder (GAD-7), perceived stress (PSS-10), and depression (PHQ-8). This allowed us to combine macrolevel variables with individuallevel variables, with a country as a grouping variable. Gender, place of residence, and education constituted individual-level predictors and were estimated at the population and country levels (i.e., these effects could vary between countries), and were entered into the model with sum-to-zero contrasts. The country-level predictors, GII, stringency, and S&P rating were recoded to provide an informal grouping of countries surveyed in the study. The GII and stringency were coded with sum-to-zero contrast, whereas linear (L) and quadratic trends (Q) were estimated for the S&P rating due to the ordinal character of the variable. Dependent variables were z-scaled before modeling; thus, the regression weights were on a standardized scale. Additionally, Bayesian *R*^2^ was provided for each model^53^. A summary of the models parameters are presented in Table 4.

**Table 4:** Regression weights from the Bayesian skew-normal multilevel models.

Parameter		GAD-7			PSS-10			PHQ-8		
		Est	LI	UI	Est	LI	UI	Est	LI	UI
β	Intercept	− 0.02	− 0.44	0.37	− 0.01	− 0.47	0.42	− 0.03	− 0.42	0.37
	Gender	**0.09**	**0.04**	**0.14**	**0.24**	**0.16**	**0.3**	**0.12**	**0.06**	**0.18**
	Residence [1]	− 0.02	− 0.1	0.05	0.01	− 0.09	0.11	**− 0.1**	**− 0.19**	**− 0.01**
	Residence [2]	0.02	− 0.05	0.1	0.07	− 0.07	0.2	0.04	− 0.05	0.14
	Residence [3]	− 0.06	− 0.12	0.01	− 0.07	− 0.19	0.04	− 0.04	− 0.13	0.05
	Education	0.04	− 0.01	0.08	0.07	− 0.04	0.17	**0.08**	**0.01**	**0.13**
	GII	− 0.05	− 0.44	0.35	− 0.21	− 0.64	0.28	− 0.1	− 0.51	0.34
	Stringency	0.04	− 0.37	0.46	0.02	− 0.45	0.53	0	− 0.4	0.41
	S&P rating [L]	− 0.15	− 0.91	0.64	− 0.31	− 1.15	0.63	− 0.26	− 1.01	0.58
	S&P rating [Q]	0.07	− 0.64	0.7	− 0.22	− 0.94	0.56	0.04	− 0.6	0.72
σ	Residual SD	0.93	0.9	0.96	0.94	0.92	0.97	0.96	0.93	0.99
α	Skewness	7.84	6.2	9.89	− 1.02	− 1.37	− 0.44	5.37	4.4	6.47
R^2^	Pop. L. Eff	0.07	0.02	0.21	0.13	0.06	0.26	0.08	0.03	0.22

Est., posterior mean; LL and UL, lower and upper bounds of the 95% credibility interval; β, standardized regression weight.

Estimations in bold are credible. Variables were coded as follows- Gender: Men = 0, Women = 1; Residence: Village = 1, Town = 2, City = 3, Agglomeration = 4; Education: Bachelor = 0, Master or higher = 1; GII: high = 0, low = 1; Stringency: high = 0, low = 1; S&P rating: 0 = low risk, 1 = medium risk, 2 = high risk (speculative); L, rating linear; Q, quadratic trends.

**Figure 3:**
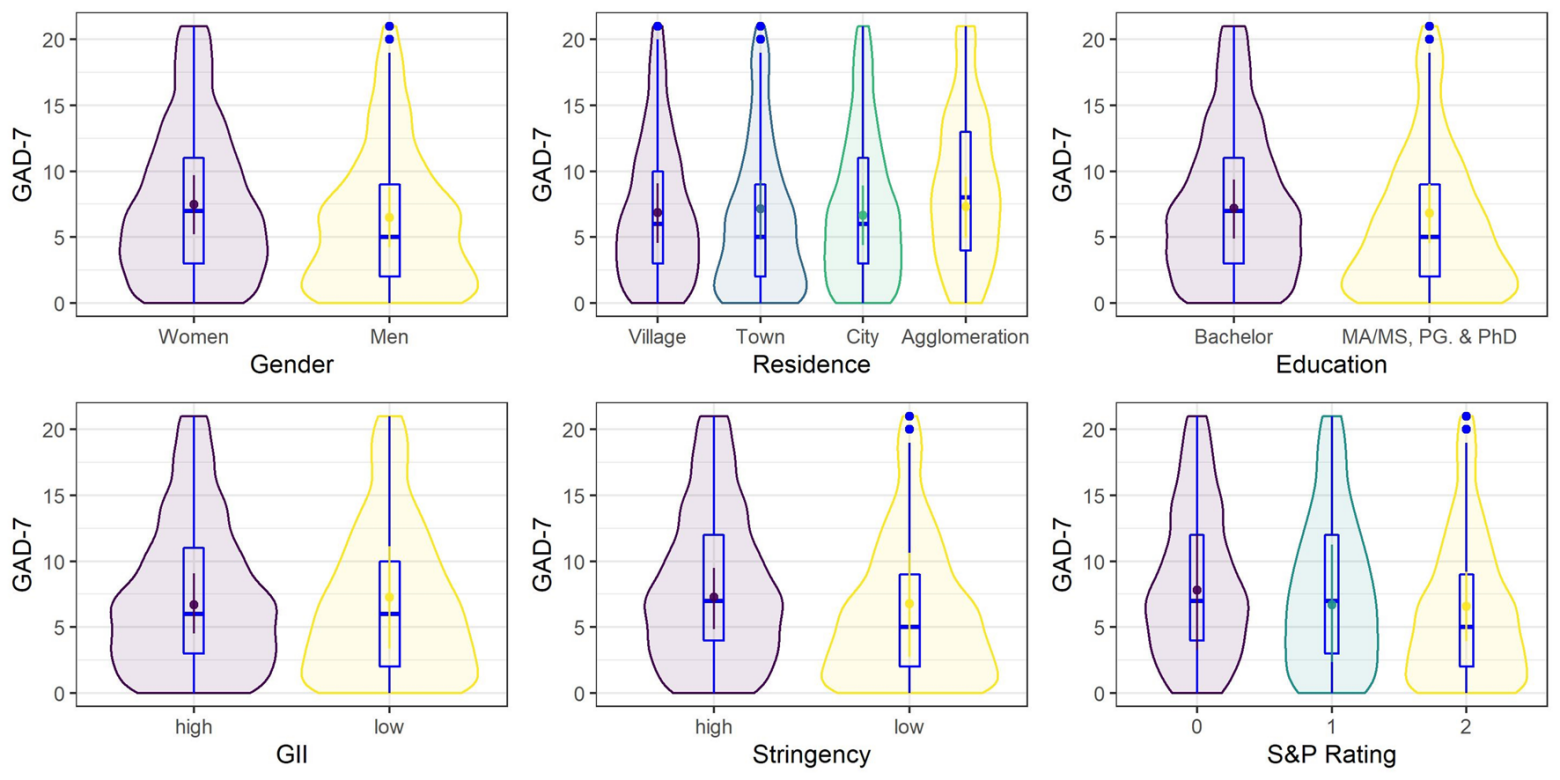
Posterior means of predicted marginal means (points) with 95% credible intervals (vertical lines) from the Bayesian skew-normal multilevel model for generalized anxiety (GAD-7) scores. Boxplots and violin plots show the distribution of the data.

**Figure 4:**
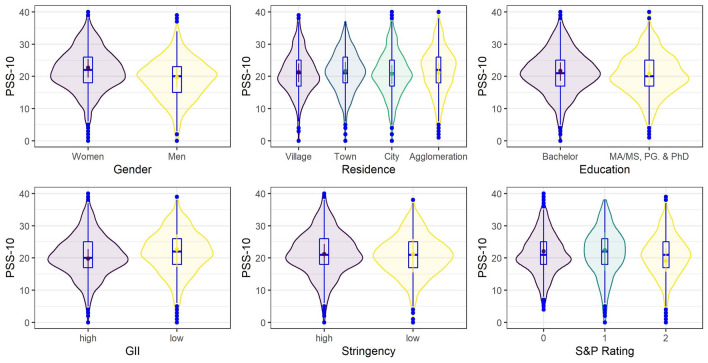
Posterior means of predicted marginal means (points) with 95% credible intervals (vertical lines) from the Bayesian skew-normal multilevel model for perceived stress (PSS-10) scores. Boxplots and violin plots show the distribution of the data.

**Figure 5:**
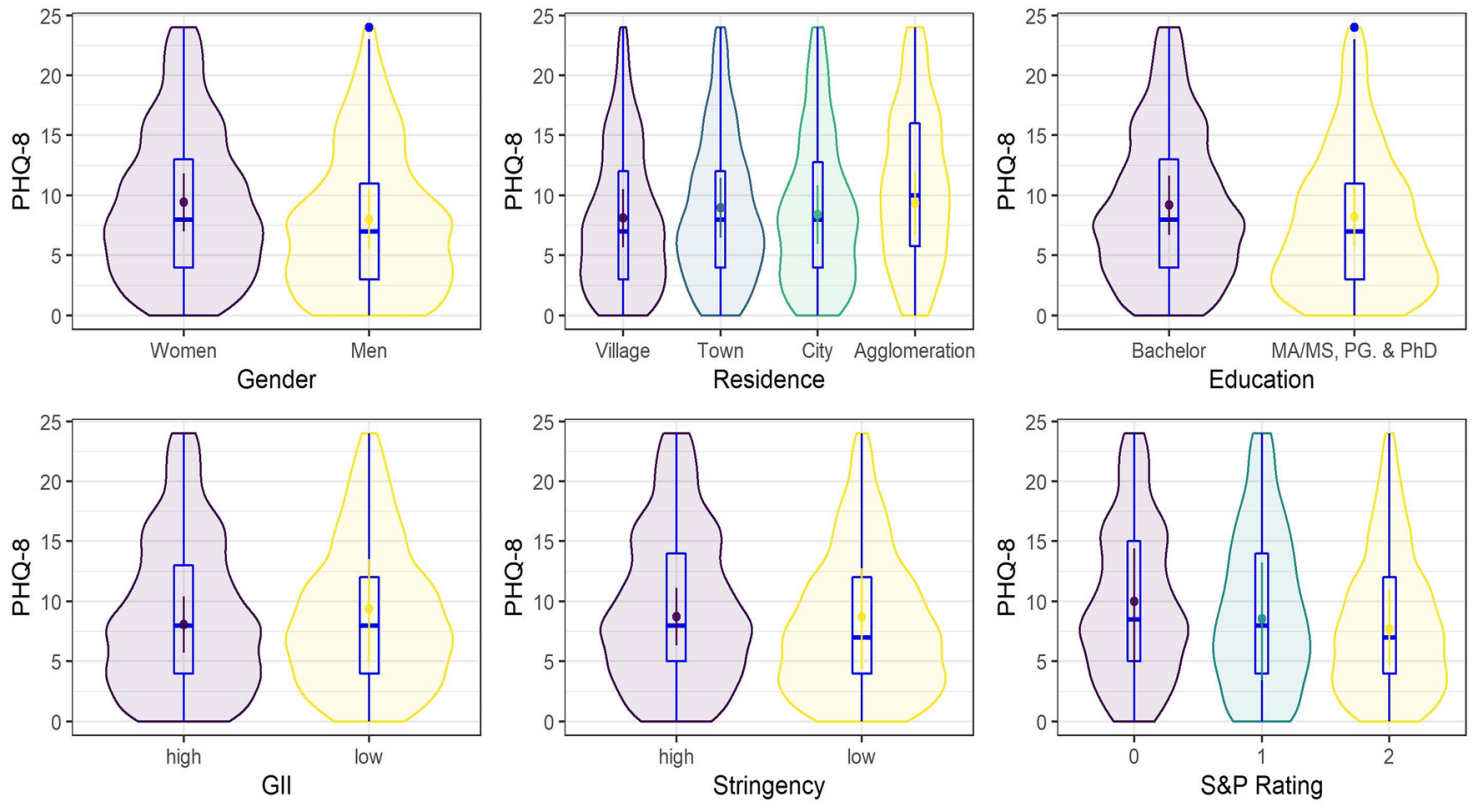
Posterior means of predicted marginal means (points) with 95% credible intervals (vertical lines) from the Bayesian skew-normal multilevel model for depression (PHQ-8) scores. Boxplots and violin plots show the distribution of the data.

Only gender had a credible effect on the GAD-7 values, with average values being credibly lower among men than among women (Fig. 3). Similarly, the average PSS-10 score was credibly lower among men than women (Fig. 4). Gender also had a credible effect on PHQ-8 scores, again with an average value credibly lower among men than among women (Fig. 5). Additionally, the average PHQ-8 was credibly lower among participants with master's degrees and higher among participants with bachelor's degrees. The effect of the place of residence was also credible. To investigate the factor further (since the regression weights represent differences from the grand mean), a post hoc analysis was conducted. The only credible difference between the averages was observed for the comparison of towns and villages, *d* = 0.14, [0.02, 0.27], with a higher average for towns (Fig. 5).

## Discussion

The present study investigated the prevalence and predictors of mental health indicators among students in nine countries during the early stage of the COVID-19 pandemic. The prevalence of high stress, depression, and generalized anxiety disorder risk among students was 61.30%, 40.3%, and 30% in the total sample, respectively. Female gender was a predictor of perceived stress, anxiety, and depression. Additionally, students living in towns (compared to those from rural areas) and those attending first cycle studies (bachelor’s students) presented a higher depression risk. However, macrolevel variables (GII, S&P rating, stringency) were irrelevant predictors of mental health.

The results of the study suggest that students in the nine countries suffered from high perceived stress and mild anxiety and depression symptoms. The international university student population experienced higher stress than the general population, which reported perceived stress at a medium level at the onset of the COVID-19 pandemic^23,26^. High stress (11.1%) and depression (6.6%) risk prevalence in the general population^26^ was substantially lower compared to the student sample in this study, at 61.30%, and 40.3%,respectively. However, a different measurement (the Multidimensional State Boredom Scale) was used in the general population study^26^. An international sample of the general population (USA and Israel) reported a similarly lower prevalence of anxiety (22.2%) (with the same cutoff point of GAD7 > 10)^54^. The prevalence of anxiety and depression risk in our study was higher than in the general Chinese population^55,56^ and a large Chinese student sample, which was 11% and 21.10%, respectively^57^. Considering that the cutoff point was lower in Chinese analyses (7) than in our research (10), the differences in depression and anxiety prevalence were even more pronounced. Our results are congruent with research showing higher anxiety rates than the general population^33,40,58^. Additionally, a recent systematic review of global prevalence of mental health issues in the general population showed prevalences of stress, depression, and anxiety, at 36.50%, 28.00%, and 26.90%, respectively^59^. In our study, students reported almost twice as high stress and depression levels.

Over half of the students in the nine countries (54.1%) did not meet any diagnostic criteria for anxiety or depression. On the other hand, 24.5% reported high depression and anxiety risk concurrently. This means that almost every fourth student in the international sample experienced the comorbidity of depression. Additionally, the prevalence of severe depression risk (12%) was almost twice as high when compared to severe anxiety prevalence risk (6.7%), which seems to be the case for the student sample^58^ but not the general population^54^.

The prevalence of anxiety (51.3%) and depression (62.3%) risk was the largest in Turkish students, whereas the lowest anxiety risk (5.2%) was in German students and the lowest depression risk (21%) in Czech students. Comparing mild anxiety symptoms among German and Russian students to the general population in those countries^25^, a similar intensity was observed in the German general population but higher (moderate levels) in the general Russian sample. However, discussion concerning the average anxiety and depression levels has been hindered by a variety of measurements used in research. On the other hand, previous evaluations of anxiety prevalence with the same measurement (GAD7) in Polish (35%)^30^ and Israeli students (56%)^58^ showed different prevalences, 46.80%, and 32.70%, respectively, compared to our results. However, those differences might have been due to the type of university or the field of study. The prevalence of moderate and high stress (84.90%) in Czech students was higher than that in the general Czech population before (35.40%) and during (51.10%) the COVID-19 pandemic^60^. Ukrainian students reported similar prevalence of anxiety and depression risk (19%) compared to a large sample of Ukrainian students in other studies during the COVID-19 pandemic (18.85%)^39^. Additionally, Polish students suffered from substantially higher depression risk prevalence (49.20%) than Polish students examined before the COVID-19 pandemic (21.98%)^61^.

The analysis of the Bayesian multilevel regression model showed that, consistent with other works^30,31,36,37^, female gender in students was a predictor of high perceived stress, anxiety, and depression. The additional predictors for depression were the place of residence and the level of study. In contrast to the results of Chinese research^40^, students from rural areas presented lower depression levels. Furthermore, students at the bachelor’s level reported higher depression than those with master's degrees or higher, which is similar to research concerning the general population^19,26^ However, being a first-cycle student was not a credible predictor of anxiety or perceived stress in the student sample. The stringency of restrictions was of trivial importance in the multilevel model. It seems that more psychological importance is laid upon the perception of restrictions' efficacy^22^, usefulness^24^, self-protective values^62^, or motives for compliance with restrictions^63^. The role of the Gender Inequality Index (GII) and global credit rating was found to be irrelevant in the surveyed models of mental health predictors. This might be due to several issues, such as the number of investigated countries. On the other hand, such a result may be explained by the specificity of the student population, which varies from the general population. For example, in our study, the number of female students exceeded that of male students in countries with high GII. Therefore, the global indices could explain mental health to a higher degree in the general population because the global student population is frequently more homogeneous.

## Limitations

There are several limitations to the present study. First, the study is of a cross-sectional nature. Second, the results pertaining to the participant level were obtained via self-reported questionnaires. Therefore, they can be subject to retrospective response bias. Finally, the lack of random sampling and the representation of the student population limited to specific regions in each country make it difficult to generalize the results. The irrelevance of macrolevel indices might be due to the small number of countries and a narrow range of indices within the nine surveyed countries. Therefore, the prospective verification of the proposed model based on a larger number of countries seems valid.

## Conclusions

The study showed that students across the nine countries seem to be a relatively homogeneous group in regard to susceptibility to mental health issues. In particular, female students are at high risk of perceived stress, anxiety and depression across Poland, Slovenia, Czechia, Ukraine, Russia, Germany, Turkey, and Colombia. As far as high perceived stress and anxiety levels are concerned, they may be interpreted as an adaptive response to the extremely volatile and unpredictable nature of the COVID-19 pandemic. Depression risk prevalence seems to be the most alarming factor, as it is even higher than the anxiety prevalence. Considering the recurring reports on severe mental health issues among students globally, the issue should be recognized at public health levels by governments or other international bodies. Our results underline the universities' need to provide dedicated programs and regular psychological support to students.

## Methods

### Study design

A cross-national study was conducted online between May and July in the following nine countries—Ukraine: 14 May–02 June, Slovenia: 14 May–26 June; Turkey: 16–29 May; Czechia: 17 June–24 July; Poland: 19 May–25 June; Israel: 21 May–03 June; Russia: 01–22 June; Germany: 02–25 June; and Colombia: 05 June.

The survey was created via Google Forms in eight countries. The German data were collected via SoSci Survey. Sampling was purposive, with the selection criterion being a university student. The invitation to the online questionnaire was sent to students by researchers via email, social media and the Moodle e-learning platform. The average time of data collection was 23.26 min (*SD* = 44.03). No form of compensation was offered as an incentive to participate. To minimize sources of bias, the student sample was highly diverse due to its key characteristics: the type of university, field of study and cycle of study.

### Ethics statement

The study protocol was approved by the University Research Committee at the University of Opole, Poland, decision no. 1/2020. The study followed the ethical requirements of the anonymity and voluntariness of participation. Each person answered the informed consent question. Following the Helsinki Declaration, written informed consent was obtained from each student before inclusion. This study is a part of an international research project: Well-being of undergraduates during the COVID-19 pandemic: International study, registered at the Center for Open Science (OSF)^64^ 10.17605/OSF.IO/BRKGD. The authors received no specific funding for this work.

### Measures

To measure whether the respondents appraised the situation in their life as stressful, the Perceived Stress Scale (PSS-10)^65^ was employed. Perceived stress is related to the subjective assessment of events occurring in one's life^66^. It evaluates how unpredictable, uncontrollable, and overloaded individuals find their lives^65^. The PPS-10 consists of 10 items referring to the frequency of stressful events that occurred in the month preceding the study, which is assessed on a 5-point scale (0 = never to 4 = very often). The Cronbach's α for this sample was 0.82.

The 7-item generalized anxiety disorder (GAD-7) scale^67^ is a self-reported measure designed to screen for symptoms following Diagnostic and Statistical Manual of Mental Disorders, fifth edition (DSM-V) criteria^68^. Based on those criteria, the generalized anxiety disorder (GAD) is characterized by persistent and excessive worry about various issues. It relates to anxiety as a state^67^. People rated how often they experienced anxiety symptoms in the 2 weeks preceding the study on a 4-point Likert scale (0 = not at all, 1 = several days, 2 = more than half the days, and 3 = nearly every day). The GAD-7 ranges from 0 to 21. Scores above 10 points indicate an anxiety disorder risk^67^. The Cronbach's α for the GAD-7 in this study equals 0.92.

The Patient Health Questionnaire (PHQ-8) was used to measure depression symptoms. The PHQ-8 consists of eight items, conforming to the DSM-V diagnostic criteria^68^. Depression is one of the most common yet treatable mental health disorders^69^. The symptoms include depressed mood, loss of interest in most or all activities, loss of energy, or feeling of worthlessness^70^. Participants use a Likert-type response scale ranging from 0 = not at all to 3 = nearly every day. The range of PHQ-8 scores is 0–24. A cutoff score of 10 or above is recommended to screen for major depressive disorder risk^70^. The individual language versions were derived from the Multicultural Mental Health Resource Centre. The internal consistency reliability of the original version measured by Cronbach's α was 0.86, and 0.88 in this study.

Demographics included four questions on gender, place of residence, level of study, and type of study (full-time vs part-time). The questionnaire was primarily designed in Polish and English, and further translated from English into Slovenia, Czech, Ukrainian, Russian, German, Turkish, Hebrew and Spanish, using backward translations by a team consisting of native speakers and psychology experts, according to the guidelines^71^. Details regarding each country and the total sample are shown in Table 1.

### Gender Inequality Index

The Gender Inequality Index (GII) measures gender inequalities in three dimensions (and five indicators) of reproductive health (maternal mortality and adolescent birth ratio), empowerment (education, parliamentary seats), and labor market (labor force participation). The GII value ranges from 0 to 1, where 0 indicates equality between women and men, while 1 indicates inequality in the aforementioned dimensions (women fare as poorly as possible). Detailed values of the GII were derived from the United Nations Human Development Programme^42^ and were recoded with respect to the median to provide an informal grouping of countries included in the study. Detailed values are presented in Table S1.

### Standard and Poor's Global Ratings

Standard and Poor's Global Ratings refers to the assessment of credit risk for investments in a particular country. It is based on a variety of factors, such as economic diversity and volatility, effectiveness, stability, and predictability of policy-making, political institutions, and civil society. Therefore, the values range from low credit risk AAA to extremely high risk at the D level. Countries in this study were divided into three groups based on the S&P rating^43^. Group A was characterized by low credit risk (Germany, Israel, Slovenia, Czechia, Poland), Group B with medium credit risk but still valid investment level (Russia and Colombia), and finally Group C at the speculative level with high credit risk (Turkey, Ukraine)—operationalized respectively as O, 1, 2. A detailed description of the S&P Global rating can be found in Table S1.

### The Oxford COVID-19 Government Response Tracker (OxCGRT)

The Oxford COVID-19 Government Response Tracker (OxCGRT) enables tracking of the stringency of government responses to the COVID-19 pandemic across countries and time^51^. The stringency level is composed of multiple indicators. It refers to community mobility: school closings, workplace closings, cancelation of public events, restrictions on gathering, public transport closings, stay at home requirements, restrictions on internal movement, international travel restrictions; and economic measures: income support, debt/contract relief, fiscal measures, and international support. The final set of indices relates to public health issues: public information campaigns, testing policy, contact tracking, emergency investment in health care, investments in vaccines, facial coverings, and vaccination. However, not all indexes were available in all countries. Those detailed measurements are rescaled to a value ranging from 0 to 100, where 100 denotes the strictest restrictions.

The timing was crucial for the stringency level evaluation. The stringency value in this study was evaluated based on the mean of the given stringency value on the first and the last days of data collection in each country^51^. Subsequently, those values for each country were dichotomized to the median to be incorporated into the statistical model. The results are shown in Table S1.

### Statistical analysis

The study used SPSS.25 (IBM, Armonk, NY, USA) and R 4.0.2 statistical software^72^. The analysis encompassed descriptive statistics: mean (M), standard deviation (SD), 95% confidence interval (CI) with lower limit (LL) and upper limit (UL). Hot-deck imputation was introduced to address a low number of missing data (n = 5, 0.02%) and they were included in the statistical analysis.

To verify the prediction model, Bayesian multilevel skew-normal regression analyses, with a country as a grouping variable (i.e., 'random effect'), were carried out using R 4.0.2 statistical software^72^. In Bayesian statistics, the inference is based on analyzing the posterior probability distributions of model parameters, obtained by integrating likelihood (data) with prior probability distributions. The parameter (e.g. regression weight) is said to be statistically credible when 95% credibility intervals (CI) of the posterior distribution exclude zero^73^. As a point estimate of the effect, the means of posterior distributions are presented. A standard normal distribution (M = 0, SD = 1) was used as a prior for the regression weights.

The approximated posterior distributions of the models were accompanied by a Markov chain Monte Carlo (MCMC) sampling procedure using the brms package^74^. For each reported model, six parallel MCMC chains were used. Each chain consisted of 8000 samples, with 4000 samples used as a warmup period and every 10th sample recorded, resulting in 2400 recorded samples in total. The sampling procedure was efficient and resulted in well-mixed and not autocorrelated chains and unimodal posteriors.

## Supplementary Information

Supplementary Information.

## Data Availability

The materials and methods are accessible at the Center for Open Science (OSF)^64^, titled: Well-being of undergraduates during the COVID-19 pandemic: International study, at 10.17605/OSF.IO/BRKGD. The datasets generated during and/or analyzed during the current study are available from the corresponding author on reasonable request.
