# Supplementary material for: Mental health prevalence and predictors among university students in nine countries during the COVID-19 pandemic: a cross-national study
Source: Sci Rep. 2021 Sep 20;11:18644. doi: 10.1038/s41598-021-97697-3 (PMC8452732; doi:10.1038/s41598-021-97697-3)
Supplement: Supplementary file 1 — Supplementary Information. [file 41598_2021_97697_MOESM1_ESM.pdf]

Supplementary Information

Mental Health Prevalence and Predictors Among University Students in Nine Countries During the COVID-19 Pandemic: A Cross-National Study

Dominika Ochnik[1], **Aleksandra M. Rogowska**[2], **Cezary Kuśnierz**[3], **Monika Jakubiak**[4], **Astrid Schütz**[5], **Marco J. Held**[5]*, **Ana Arzenšek**[6], **Joy Benatov**[7], **Rony Berger**[8,9], **Elena V. Korchagina**[10], **Iuliia Pavlova**[11], **Ivana Blažková**[12], **Imran Aslan**[13], **Orhan Çınar**[14,15], **Yonni Angel Cuero-Acosta**[16]

[1]University of Technology, Faculty of Medicine, Katowice, 40-555, Poland.

[2]University of Opole, Institute of Psychology, Opole, 45-052, Poland.

[3]Opole University of Technology, Faculty of Physical Education and Physiotherapy, Opole, 45-758, Poland.

[4]Maria Curie-Sklodowska University in Lublin, Faculty of Economics, Lublin, 20-031, Poland.

[5]University of Bamberg, Department of Psychology, Bamberg, 96047, Germany.

[6]University of Primorska, Faculty of Management, Koper, 6101, Slovenia.

[7]University of Haifa, Department of Special Education, Haifa, 3498838, Israel.

[8]The Center for Compassionate Mindful Education, Tel Aviv, 69106, Israel.

[9]Tel-Aviv University, Bob Shapell School of Social Work, Tel Aviv, 69978, Israel.

[10]Peter the Great St. Petersburg Polytechnic University, Institute of Industrial Management, Economics and Trade, St. Petersburg, 195251, Russia.

[11]Lviv State University of Physical Culture, Department of Theory and Methods of Physical Culture, Lviv, 79007, Ukraine.

[12]Mendel University in Brno, Department of Regional and Business Economics, Brno, 613 00, Czech Republic.

[13]Bingöl University, Health Management Department, Bingöl, 12000, Turkey.

[14]Ataturk University, Faculty of Economics and Administrative Sciences, Erzurum, 25240, Turkey.

[15]Ağrı İbrahim Çeçen University, Faculty of Economics and Administrative Sciences, Ağrı, 04000, Turkey.

[16] Universidad del Rosario, School of Management, Bogotá, PC 111711, Colombia.

*marco.held@uni-bamberg.de

Table S1. Values and levels of the COVID-19 pandemic restrictions' stringency (Stringency) and socio-economic indexes: Gender Inequality Index (GII) and S&P Global Rating in the nine countries.

Country		Stringency	GII	S&P Rating	Stringency	GII	S&P Rating
		Values			Levels		
	Range	0-100	0-1	AAA-D	0-1	0-1	0-2
Poland		67.13	.12	A-	0 low	0 low	0 low
Slovenia		47.91	.07	AA-	0 low	0 low	0 low
Czechia		41.67	.14	AA-	0 low	1 high	0 low
Ukraine		82.64	.29	B	1 high	1 high	2 high
Russia		74.54	.25	BBB-	0 low	1 high	1 medium
Germany		61.57	.08	AAA	1 high	0 low	0 low
Turkey		75.93	.30	B+	1 high	1 high	2 high
Israel		75.00	.10	AA-	1 high	0 low	0 low
Colombia		87.04	.41	BBB-	1 high	1 high	1 medium

Note: S&P Global Rating index refers to credit risk estimation.

Supplementary Note

The recruitment details of the university students from Poland, Slovenia, Czechia, Ukraine, Russia, Germany, Turkey, Israel, and Colombia

The Polish sample consisted of 301 students recruited from Maria Curie-Sklodowska University (UMCS) in eastern Poland (n = 149, 48%) and from University of Opole in the south of Poland (n = 152, 51%). In Slovenia, all students were from University of Primorska in Koper (n = 209, 100%), in Czechia from Mendel University in Brno (n = 310, 100%), and in Ukraine from Lviv State University of Physical Culture (n = 310, 100%). Russian students were from universities located in Sankt Petersburg: Peter the Great St. Petersburg Polytechnic University (n = 155, 54%), HSE University (n = 90, 31%), and St. Petersburg State University of Economics and Finance (n = 42, 15%). The German sample was from University of Bamberg (n = 270, 100%) whereas the Israeli sample was from University of Haifa (n = 199, 100%). Turkish students were from eleven Turkish universities mostly located in eastern Turkey: Bingol University, Bingöl (n = 148, 48%); Atatürk University, Erzurum (n = 110, 35%); Muğla Sıtkı Koçman University, Muğla (n = 35, 11%); Ağrı İbrahim Çeçen University, Ağrı (n = 6, 2%); Fırat University, Elazığ (n = 3, 0.8%); Kırıkkale University, Kırıkkale (n = 1, 0.3%); Adnan Menderes University, Aydın (n = 1, 0.3%); Başkent University, (n = 3, 1%); Boğaziçi University (n = 1, 0.3%), Dicle University, Diyarbakır (n = 1, 0.3%), and Istanbul University (n = 1, 0.3%). Colombian students (n = 155) were recruited from Bogota universities: Del Rosario University (n = 142, 92%) and El Bosque University (n = 13, 8%).
